# Supplementary material for: Remote Sensing Evaluation Drone Herbicide Application Effectiveness for Controlling Echinochloa spp. in Rice Crop in Valencia (Spain)
Source: Sensors (Basel). 2024 Jan 25;24(3):804. doi: 10.3390/s24030804 (PMC10857354; doi:10.3390/s24030804)
Supplement: Supplementary file 1 [file sensors-24-00804-s001.zip › sensors-2801561-supplementary.pdf]

Supplementary material

The higher intensity of *Echinochloa* spp. plant damage in the control subplots is shown in Figure S1. It is observed that in the treated areas the intensity of the green color is lower.

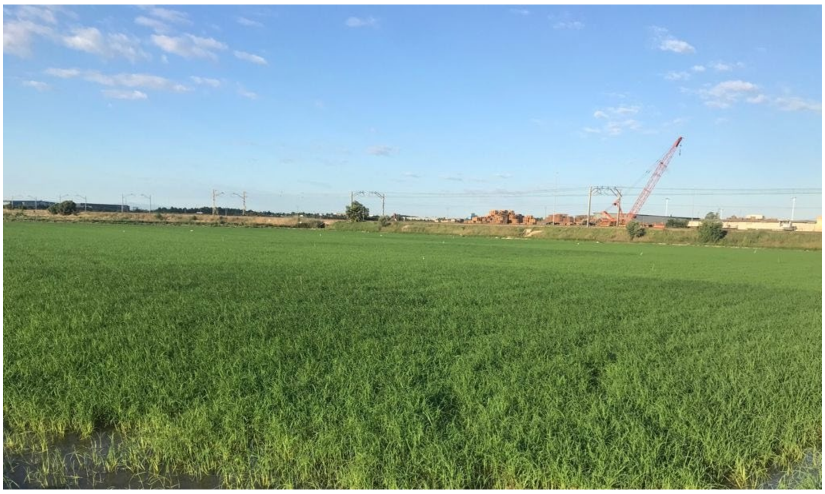

Figure S1. Control subplots affected by *Echinochloa* spp. (Own elaboration)
